# The associations of objective, behaviorally measured hunger and weight-related variables with Food Craving Inventory subscales in adults with obesity

**DOI:** 10.1038/s41366-025-01925-z

**Published:** 2025-10-04

**Authors:** William R. Quarles, Alexis Brown, Martin Binks

**Affiliations:** 1 Office of New Drugs, Food and Drug Administration, Silver Spring, MD USA; 2 Physician Assistant Studies, University of Arkansas for Medical Sciences, Little Rock, AR USA; 3 Department of Nutrition and Food Studies, George Mason University, Fairfax, VA USA

**Keywords:** Nutrition, Translational research, Obesity

## Abstract

**Background/Objectives:**

The Food Craving Inventory (FCI) measures the frequency of food cravings along five dimensions: overall food cravings, cravings for sweets, cravings for high-fat food, cravings for starchy food, and cravings for fast food. Evidence of the influence of current hunger on FCI scores is equivocal and challenged by methodological limitations, including the use of self-reported hunger and the lack of control for phase of the menstrual cycle. We aimed to examine (1) the potential association of objective, behaviorally measured hunger (hours since last caloric intake; FAST) with responses on the FCI, controlling for the influence of the menstrual cycle and (2) the relationships of the FCI with weight-related measures (i.e., body mass index, BMI; body weight, BW; body fat percentage, BF) in individuals with obesity.

**Subjects/Methods:**

Thirty-two adults with obesity (BMI 30–39.9 kg/m^2^) were included. Subjects’ BW and BF were measured via bioelectrical impedance, and BMI was calculated. On a separate day, subjects were administered the FCI after an intended 8-h fast. For premenopausal women, this was during the second half of the follicular phase of their menstrual cycle. Linear correlations between FCI subscales and each of BW, BMI, BF and FAST were performed.

**Results:**

FAST correlated with cravings for sweets (*r* = 0.381, *p* = 0.034) and showed a trend for overall cravings (*r* = 0.331, *p* = 0.069). BW, BMI, and BF did not correlate with any of the FCI subscales.

**Conclusions:**

Our results show that current, objective, behaviorally defined hunger (FAST) may influence FCI scores after controlling for menstrual cycle phase. However, FCI scores showed no relationship to BMI, BW, or BF, indicating that FCI scores may not reflect enduring patterns related to long-term weight gain. Future research should use objective hunger measures as covariates when interpreting FCI data and should exercise caution in treating FCI scores as indicators of long-term obesity risk.

## Introduction

Food cravings are complex psychological phenomena characterized as frequent, intense desires to consume a specific type of food that is often difficult to resist. The Food Craving Inventory (FCI) is a validated self-report questionnaire designed to quantify the frequency of food cravings along five dimensions: overall food cravings (FCI-O); cravings for sweets (FCI-S); cravings for high-fat food (FCI-H); cravings for starchy food (FCI-St); and cravings for fast food (FCI-FF) [1]. Specific food craving categories, as measured by the FCI, are positively associated with food intake of the same category at a meal [2, 3], as well as habitual intake of food of that type [4]. The construct validity of the FCI is well established in studies demonstrating positive associations between scale-specific cravings and specific food intake [2, 5, 6].

Several potential neurobiological mechanisms underlying these hunger-craving associations are suggested by the literature. Extended fasting periods have been shown to modulate neural responses to food cues in reward-related brain regions [7, 8]. Moreover, an objective measure of homeostatic hunger (i.e., plasma insulin) was more closely aligned with neural activation of reward-related brain regions than subjective hunger ratings [9]. Furthermore, changes in ghrelin have been positively correlated with a six-item FCI “state” scale (pizza, brownie, chocolate, cookies, ice cream, steak), and higher baseline ghrelin is predictive of longitudinal increases in FCI-St and FCI-O after adjusting for demographic variables, baseline BMI, and baseline food cravings [10]. Finally, recent studies have shown that treatments involving tirzepatide, a GIP/GLP-1 analog, reduce FCI-O, FCI-S, and FCI-St in people with obesity [11]. These findings collectively suggest potential mechanistic underpinnings of FCI outcomes.

It is in this that we note two significant limitations in the current FCI literature. First, the majority of clinical studies that use the FCI, in their attempts to control for the influences of hunger, rely on self-reported hunger (i.e., visual analog scales; VAS) [12, 13] rather than objective, behaviorally defined measures, which may be more appropriate in the context of the neurohormonal influences described above. This is further supported by the fact that self-reported hunger reflects the psychological drive to eat, which is distinct from food cravings and physiological hunger [13]. Together, these findings suggest that the typical practice of using VAS to measure hunger [14] may not be optimal in light of the likely impact of objective hunger states on the FCI. This further suggests that the use of objectively measured, behaviorally defined hunger (hours since last caloric intake; FAST) may be preferable. Further support for this notion can be found elsewhere in the literature. Objective hunger (i.e., a 4-h fast) has been found to affect image-induced food cravings [15], and previous studies have suggested that food cravings may develop as a result of learned associations between ingesting specific foods and hunger states (a 2-h fast vs. a fed state) [16]. Importantly, objective hunger (i.e., an 18-h fast) increases neural activity response to high-calorie foods [17], suggesting that hunger may increase the likelihood of energy-dense food cravings. Additional evidence further suggests that objective hunger (i.e., a 17-h fast) can make food cravings more difficult to resist [18]. Despite these findings, most FCI studies either neglect to quantify current hunger or rely on unreliable [19] self-report modalities (e.g., VAS).

In summary, the convergence of evidence suggests that hunger may modulate FCI scores and that to accurately capture hunger as a covariate in FCI studies, behaviorally defined, objectively measured hunger (FAST) may be the most appropriate method.

Another limitation in the current literature is that few studies adequately account for menstrual cycle phase, despite its known influences on subjective food cravings. Neuroimaging studies have found that the phase of the menstrual cycle can have salient influences on food cue reactivity in premenopausal women as measured by functional magnetic resonance imaging (fMRI-FCR) [20, 21], where incidental characteristics are crucial to control for [22]. Furthermore, certain phases of the menstrual cycle have been associated with consumption of sweet, carbohydrate, and fatty foods [23, 24]. Together, these suggest that it is imperative to control for menstrual cycle influences when measuring food cravings with the FCI.

A final and important consideration when using the FCI lies in the interpretation of the FCI as representing an enduring pattern in the relationship with food, versus a more transient, situationally specific affinity [21]. Evidence supporting the FCI as a reflection of an enduring pattern that contributes to longer-term weight gain via chronic over-ingestion patterns remains equivocal. In one cross-sectional study, each of the FCI subscales was found to have weak, positive associations with BMI (FCI-S: *r* = 0.13, *p* < 0.05; FCI-H: *r* = 0.21, *p* < 0.001; FCI-St: *r* = 0.15, *p* < 0.001; FCI-FF: *r* = 0.13, *p* < 0.05) [4]. However, this study had a large sample size (*n* = 646); and the variance of BMI accounted for by each of the FCI constructs was low (FCI-S: 1.7%; FCI-H: 4.4%; FCI-St: 2.3%; FCI-FF: 1.7%), suggesting a relatively low effect size compared to other, more salient predictors. In another cross-sectional worksite study, it was found that FCI-O was associated with BMI (*r* = 0.21, *p* = 0.046), with similarly weak associations noted [25]. The variance explained was similarly less than 5%. Together, these findings do not present compelling evidence that food cravings, as measured by the FCI, represent an enduring propensity towards ingestion based on the presence of food cravings.

In summary, while the FCI is widely used in the obesity and ingestive behavior literature to better understand the psychological and neurophysiological response to dietary interventions [26, 27], there are methodological considerations that warrant attention. Here we seek to examine (1) the potential association of objective, behaviorally measured hunger (FAST) with responses on the FCI, controlling for the influence of the menstrual cycle and (2) the relationships of the FCI with weight-related measures (i.e., BMI, BW, BF) in a treatment-seeking population with class I & II obesity.

## Methods

### Subjects

Thirty-two adults (ages 19–60 years; M = 14, F = 18) with obesity (BMI 30–39.9 kg/m^2^) who completed the initial assessment as part of a larger randomized clinical trial [28] were included. Exclusion criteria for the larger trial were: having a motor, visual or hearing impairment or any contraindications for fMRI scanning (e.g., an implanted medical device, any ferrous metal in the body); diagnosed diabetes mellitus, uncontrolled hypertension, history of ischemic heart disease, cerebrovascular accidents, neurological disease, or current severe psychiatric illness. Female subjects were excluded if they had an irregular menstrual cycle, were pregnant or were attempting to conceive.

### Procedures

Potential subjects were screened for eligibility by telephone before attending an in-person session. During the in-person session, height was measured using a stadiometer (HR-200 TANITA wall-mounted height rod, TANITA Corporation of America Inc., IL, USA), and BW, BF, and BMI were measured via bioelectrical impedance analyzer (TANITA BC-418 segmental body composition analyzer; TANITA Corporation of America Inc., IL, USA), as part of the determination for study eligibility. On a separate day, subjects were administered the FCI after an intended 8-h fast, before undergoing their baseline fMRI scans in the larger study. For premenopausal women, this was during the second half of the follicular phase of their menstrual cycle (i.e., day 10–14). This allowed for scheduling visit 5 within the follicular phase of the subsequent menstrual cycle (i.e., approximately day 3–7) to avoid menstrual influences on fMRI-FCR in the larger study [27].

### Objective, behaviorally defined hunger (i.e., hours since last caloric intake; FAST)

For the purposes of this study, hunger was defined as hours since “last meal” which was further defined as “any caloric consumption including caloric beverages.”

### Food Craving Inventory (FCI)

The FCI is a 37-item questionnaire that measures food cravings for sweets, high-fat foods, starchy foods, fast food, and overall cravings. Subjects are asked, “over the past month, how often have you experienced a craving for the food?” and to rate each food from “Never” to “Always / almost every day” on a 5-point Likert scale. The scores are summed and the items for each food category are averaged into the total score for each factor. The FCI has acceptable internal consistency (Cronbach’s alpha 0.76–0.93) and reasonable test-retest reliability (0.79–0.91).

### Statistical analyses

All analyses were performed using R statistical software (version 3.3.2) [29]. One subject was excluded from the analysis for not following the study visit preparation instructions. Linear correlations were examined between all subscales of FCI and BW, BMI, and BF separately. An exploratory analysis was performed to examine the linear correlations between subscales of FCI and the actual duration of fasting (FAST) prior to FCI administration.

## Results

Data for 31 individuals with obesity (mean BMI: 34.74 ± 3.26 kg/m^2^; mean age: 31.45 ± 15.55 years) were included in the analyses. BW, BMI, and BF did not significantly correlate with any subscales (Table 1). However, FAST correlated with cravings for sweets (*r* = 0.381 [95%CI 0.031, 0.648], *p* = 0.034) and approached significance for overall cravings (*r* = 0.331 [95%CI −0.027, 0.613], *p* = 0.069) and cravings for fast food (*r* = 0.304 [95%CI −0.055, 0.595], *p* = 0.095).

**Table 1:** Associations between FCI subscales and body weight, body fat, body mass index, and fasting duration.

Relationship	Pearson’s *r*	*p*	95% CI
BW & FCI-O	0.176851414	0.341232177	[−0.18935, 0.49987]
BW & FCI-S	0.23723843	0.19877657	[−0.12785, 0.5457]
BW & FCI-H	0.294719443	0.107512437	[−0.06657, 0.58769]
BW & FCI-St	−0.024185557	0.897243378	[−0.37531, 0.33301]
BW & FCI-FF	0.032577915	0.861882629	[−0.32552, 0.3825]
BF & FCI-O	0.153022599	0.411168884	[−0.21286, 0.48127]
BF & FCI-S	0.112254689	0.547687655	[−0.25211, 0.44875]
BF & FCI-H	0.03635423	0.84605235	[−0.32214, 0.38573]
BF & FCI-St	0.228894971	0.215499176	[−0.13652, 0.53948]
BF & FCI-FF	0.072149961	0.699712047	[−0.28959, 0.41586]
BMI & FCI-O	0.148444283	0.42546936	[−0.21733, 0.47766]
BMI & FCI-S	0.188415051	0.310072979	[−0.1778, 0.50879]
BMI & FCI-H	0.084816299	0.650080817	[−0.27788, 0.42634]
BMI & FCI-St	0.046116194	0.80541636	[−0.31334, 0.39402]
BMI & FCI-FF	−0.045407423	0.808351376	[−0.39342, 0.31398]
FAST & FCI-O	0.330587106	0.069301716	[−0.0269, 0.61311]
FAST & FCI-S	0.38145726	0.034226668	[0.03136, 0.64819]
FAST & FCI-H	0.238995219	0.19537334	[−0.12602, 0.54701]
FAST & FCI-St	0.262346444	0.153934372	[−0.10142, 0.56424]
FAST & FCI-FF	0.304877398	0.095375162	[−0.05545, 0.59495]

## Discussion

Findings provide preliminary evidence that objective, behaviorally measured hunger (i.e., FAST) is associated with scores on FCI subscales in a treatment-seeking population with class I & II obesity. Specifically, FAST was associated with FCI-S, and approached significance for associations with FCI-O, FCI-FF, explaining approximately 14%, 11%, and 9% of the variance of the subscales, respectively. These associations are notably stronger than those reported in larger cross-sectional studies that relied solely on self-reported hunger [30] and did not control for the phase of the menstrual cycle. The relatively larger proportion of explained variance in this study suggests that the current state of hunger plays a more substantial role in FCI scores than previously thought when using a more robust study design (i.e., behaviorally defined hunger as opposed to self-report; controlling for menstrual phase). These relatively larger effect sizes suggest that implementing more rigorous methodological controls may reveal stronger hunger-craving associations than previously recognized in the literature.

These findings have important implications for the measurement and interpretation of food cravings in clinical and research contexts. The stronger explanatory power of objective fasting duration, compared to traditional VAS, suggests that objective, quantitative measures of hunger may provide more reliable covariates when utilizing the FCI. These results corroborate findings that objective hunger measurements are more tightly linked to attenuated activation in reward-related brain regions than subjective hunger ratings [9].

The absence of significant associations in the relationship between FCI and weight-related variables also merits careful consideration in the context of FCI use and interpretation. These findings, consistent with previous large-scale studies reporting weak correlations between body weight-related variables and FCI [31], suggest that food cravings as measured by FCI do not represent stable factors that are predictive of long-term weight trajectory. This conclusion is particularly significant given our robust methodological control for menstrual cycle phase, suggesting that even when controlling for influences of the menstrual cycle, FCI subscales may not be representative of an enduring pattern that contributes to long-term weight gain.

### Strengths and limitations

The study has methodological strengths that improve upon the existing literature and inform future studies. First, the experimental design controlled the phase of the menstrual cycle. Given the known relationship of food craving to the menstrual cycle, managing this confounding variable is essential. Furthermore, managing it prospectively in the experimental design is a more robust approach. Second, the study excluded individuals with diabetes mellitus [32], which is important because diabetes mellitus can confound results due to hormonal influences on craving. Third, the study quantified hunger objectively by using the number of hours since last caloric intake, eliminating the confluence of psychological state with objective hunger that is created via self-reported hunger.

There are also some limitations. First, the current study employed a restricted BMI range (Class I & II obesity only), limiting the generalizability to other BMI categories. Additionally, the study was not designed to test the stability of the FCI factors themselves; instead, it relied only on how they are related to known outcomes. A future study could utilize a longitudinal design with equal control to explore the stability of the factors themselves. Third, we did not standardize the macronutrient content or energy density of the evening meal that preceded the overnight fast. Fourth, we did not control for the use of oral contraceptives, which may further influence FCI scores due to their effect on cyclical sex-hormone fluctuations. However, given that oral contraceptives stabilize hormone levels by suppressing fluctuations characteristic of the natural menstrual cycle, it is unlikely that this confounded our findings to a significant degree.

### Summary and conclusions

These findings suggest that scores on the FCI may be influenced by current, objective, behaviorally defined hunger (FAST) when controlling for phase of the menstrual cycle. Future studies should consider objective, behaviorally defined hunger (i.e., time since last caloric consumption) as a covariate. Findings further suggest that current craving, as measured by FCI, may not be representative of a more enduring pattern that contributes to longer-term weight gain.

## Data Availability

Data described in the manuscript, code book, and analytic code will be made available upon request.
